# FXR activation promotes intestinal cholesterol excretion and attenuates hyperlipidemia in SR‐B1‐deficient mice fed a high‐fat and high‐cholesterol diet

**DOI:** 10.14814/phy2.14387

**Published:** 2020-03-13

**Authors:** Amar B. Singh, Bin Dong, Fredric B. Kraemer, Jingwen Liu

**Affiliations:** ^1^ Veterans Affairs Palo Alto Health Care System Palo Alto CA USA; ^2^ Department of Medicine Stanford University School of Medicine Stanford CA USA

**Keywords:** bile acid, cholesterol transporter, fecal cholesterol, FXR, obeticholic acid, SR‐B1

## Abstract

Obeticholic acid (OCA) activates the farnesoid X receptor (FXR) to lower circulating total cholesterol (TC) and high density lipoprotein‐cholesterol (HDL‐C) concentrations and to stimulate fecal cholesterol excretion in mice by increasing hepatic SR‐B1 expression. Here we show that hepatic SR‐B1 depletion by an adenovirus expressing Sr‐b1 shRNA (Ad‐shSR‐B1) attenuated these beneficial effects of OCA in mice on a chow diet. The mRNA levels of ABC cholesterol transporter genes (Abca1, Abcg1, Abcg5, and Abcg8) were unchanged in the liver of hepatic SR‐B1‐depleted mice regardless of OCA treatment; however, a modest increase in Abca1, Abcg5, and Abcg8 mRNA levels was observed in the ileum of vehicle‐treated control mice and Abca1 and Abcg8 mRNA levels were increased more by OCA administration. OCA treatment of Sr‐b1 knock out (KO) mice (Sr‐b1‐/‐) fed a normal chow diet (NCD) displayed a similar lack of transhepatic cholesterol movement, as well as a modest increase in the levels of ileum cholesterol transporter expression. However, OCA treatment of Sr‐b1 KO mice fed a cholesterol‐enriched diet reduced circulating cholesterol and increased fecal cholesterol output to comparable degrees to that of wild‐type (WT) mice, and these effects were accompanied by substantial elevations of mRNA levels of Abca1, Abcg1, Abcg5, and Abcg8 in the ileum of Sr‐b1 KO mice. Our studies suggest that FXR activation stimulates intestinal cholesterol excretion and reduces diet‐induced hyperlipidemia through increased expression of ileal cholesterol transporters when hepatic SR‐B1‐mediated cholesterol movement is absent.

## Graphical abstract

FXR activation in Srb1 KO mice fed a cholesterol‐enriched diet increased liver LDL receptor expression and stimulated the gene expression of cholesterol transporter in ileum, which let to the substantial reduction of serum cholesterol and the increased fecal cholesterol output.

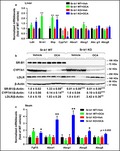

## 1 INTRODUCTION

The farnesoid X receptor (FXR) is a nuclear hormone receptor that is activated by bile acids (BAs) and highly expressed in the liver and other tissues (Forman et al., 1995). FXR is involved in regulating BA synthesis, secretion, absorption, and uptake by hepatocytes (Lee, Lee, Hubbert, Edwards, & Zhang, 2006). Control of the classical BA pathway is mainly regulated by the activity of cholesterol 7α‐hydroxylase (CYP7A1) and sterol 12α‐hydroxylase (CYP8B1), rate‐limiting enzymes whose transcription is inhibited by FXR‐mediated upregulation of small heterodimeric partner (NR0B2, SHP) (Lu et al., 2000), (Kong et al., 2012). In intestine, FXR activation promotes the production of fibroblast growth factor 15/19 (FGF‐19 in human), which, in turn, inhibits bile salt synthesis in the liver (Inagaki et al., 2005; Kong et al., 2012), and increases the flux of cholesterol into the intestinal lumen by activating ABCG5/ABCG8 cholesterol exporter (de Boer et al., 2017). In addition, activation of hepatic FXR modulates the expression of many hepatic genes involved in lipoprotein metabolism, including scavenger receptor class B type 1 (SR‐B1), ApoC‐II, ApoC‐III, and ApoA‐I (Lee et al., 2006; Zhang et al., 2006) and induces high‐density lipoprotein‐mediated transhepatic cholesterol efflux in mice and monkeys (Hambruch et al., 2012). Fxr^‐/‐^ mice exhibit elevated levels of plasma total cholesterol (TC) and HDL‐cholesterol (Lambert et al., 2003). In contrast, FXR ligand treatment reduces plasma HDL‐cholesterol and increases reverse cholesterol transport (RCT) by increasing the expression of hepatic SR‐B1 (Zhang, Hagedorn, & Wang, 2011). FXR activation in rodents has also been shown to protect from atherosclerosis (Hartman et al., 2009) and nonalcoholic fatty liver disease (NAFLD) (Kong, Luyendyk, Tawfik, & Guo, 2009; Zhang, Wang, Liu, & Harnish, 2009). Because of all these beneficial effects, FXR has been considered an attractive therapeutic target for the treatment of NAFLD and other liver metabolic diseases (Mudaliar et al., 2013).

SR‐B1 functions as a receptor for HDL, facilitating the selective uptake of cholesteryl esters from circulating lipoproteins, including HDL (Shen, Azhar, & Kraemer, 2018), and playing important roles in transhepatic cholesterol efflux and the regulation of high density lipoprotein‐cholesterol (HDL‐C) metabolism (Yancey et al., 2000). SR‐B1 is most highly expressed in liver and steroidogenic tissues (Landschulz, Pathak, Rigotti, Krieger, & Hobbs, 1996; Stangl, Hyatt, & Hobbs, 1999). In support of its roles in these processes, hepatic overexpression of SR‐B1 in mice markedly reduces plasma HDL‐C levels and increases biliary secretion of cholesterol (Kozarsky, Donahee, Glick, Krieger, & Rader, 2000; Kozarsky et al., 1997). Conversely, genetic knockout of Sr‐b1 in mice raised circulating HDL‐C and suppressed biliary cholesterol excretion into feces (Rigotti et al., 1997; Van Eck et al., 2003). Additionally, several studies in mice have suggested an inverse relationship between SR‐B1 expression and atherosclerosis, which appears to be principally mediated by accelerating RCT (Fuller et al., 2014; Van Eck et al., 2003).

Obeticholic acid (OCA) is a synthetic BA and FXR agonist was developed for treating primary biliary cirrhosis, nonalcoholic steatohepatitis, and other chronic liver diseases. OCA treatments have been observed to result in reductions in plasma TC, HDL‐C, and, in some cases, low density lipoprotein‐cholesterol (LDL‐C) in various animal models (Gardes et al., 2011; Singh et al., 2018; Stangl et al., 1999). Previously, we reported that OCA treatment increased hepatic SR‐B1 expression, resulting in lower plasma TC and HDL‐C levels and increases in cholesterol fecal excretion in hamsters (Singh et al., 2018). In addition, activation of FXR by OCA is linked to increased transhepatic cholesterol efflux mediated through SR‐B1 pathway in mice (Hambruch et al., 2012). Despite the established connection of increased SR‐B1 expression and enhanced transhepatic cholesterol excretion upon FXR activation by OCA or other FXR agonists, up to today, no studies have investigated the impact of SR‐B1 deficiency on FXR‐mediated regulation of plasma and hepatic lipid metabolism. In this work, we examine the impacts of FXR activation by OCA on transhepatic cholesterol elimination in global and liver‐specific Sr‐b1‐deficient mouse models under normal and hyperlipidemic conditions. Our results revealed a surprising finding that, in the absence of SR‐B1, FXR activation in the setting of a high‐fat/high‐cholesterol diet still reduced serum cholesterol and increased fecal cholesterol elimination and this occurred through the upregulation of cholesterol transporter gene expression in the ileum, a response which appeared to compensate for the loss of SR‐B1‐facilitated transhepatic cholesterol movement.

## 2 MATERIALS AND METHODS

OCA, generously provided by Mark Young (Intercept Pharmaceuticals), was suspended in 0.5% carboxymethyl cellulose (CMC) vehicle control. All reagents were purchased from Sigma unless otherwise noted.

### 2.1 Generation of liver‐specific SR‐B1‐deficient mice

All animal studies were approved by the Institutional Animal Care and Use Committee at Veterans Affairs Palo Alto Health Care System (VAPAHCS) and were consistent with the National Institutes of Health Guide for the Care and Use of Laboratory Animals. All mice were housed in temperature‐ and humidity‐controlled rooms with 12 hr light/12 hr dark cycle. Male C57BL/6J mice (6–7 weeks old) were purchased from Jackson laboratory and were maintained on sterilized standard chow diet (Cat. # AIN‐93G, Research Diets). For viral gene delivery, mice fed a normal chow diet (NCD) (D10012G, Research Diets) were given 6 × 10^11^ adenoviral particles of Ad‐shSR‐B1 (*n* = 12) or control Ad‐shU6‐C (*n* = 12) by retro‐orbital administration under isoflurane‐induced anesthesia. Seventy‐two hours postinfection, mice were divided in four groups (*n* = 6 per group) and were treated with either OCA or vehicle for 10 days. After 10 days posttreatment, mice were sacrificed, and blood, liver, gallbladder, and ileum tissues were collected and immediately flash frozen. Throughout the study, food intake and body weights were recorded.

### 2.2 Sr‐b1 KO mice breeding and treatments

Heterozygous B6/129S‐Srb1^tm1kri29^ breeding pairs were obtained from Jackson Laboratory to generate a colony. As female Sr‐b1 knock out (KO) mice are infertile (Trigatti et al., 1999), the colony was maintained by breeding homozygous (‐/‐) KO or heterozygous (±) male mice with heterozygous (±) females. Wild‐type (WT), heterozygous, and Sr‐b1 KO mice were verified by PCR amplification using a common primer (5′‐TCAAACCCTGTGACAACAGC‐3′) in combination with either mutant‐specific primer (5′‐ATAGATTCGCCCTTGTGTCC‐3′) or WT‐specific primer (5′‐ATCTCAGCCTTAGGCCCTGT‐3′). The PCR product was fractionated on 2% agarose gels and visualized with ethidium bromide. The genotype was confirmed by identifying correct bands (140 bp‐Mutant, 140 bp, 262 bp‐Heterozygote, and 262 bp‐Wild type). The animals were fed a standard chow diet and drinking water. In the first experiment, 7–8 weeks old 50% male and 50% female Sr‐b1 KO mice fed a NCD were divided into two groups (*n* = 8, 4M/4F each), and orally gavage OCA (40 mg kg^−1^ day^−1^) or vehicle for 2 weeks. At the end of the treatment, mice were fasted for 4 hr before collecting blood and tissue samples.

In another experiment, 8‐week‐old male Sr‐b1 KO mice (*n* = 8) and their WT littermate (*n* = 8) were fed a high‐fat and high‐cholesterol diet (HFHCD) containing 0.5% cholesterol (D12107, Research Diets) for 2 weeks prior to treatment with OCA (40 mg kg^−1^ day^−1^, *n* = 4) or vehicle (0.5% CMC, *n* = 4) for 2 weeks. At the end of OCA treatment, mice were sacrificed after 4 hr fasting for the collection of blood and tissue samples.

### 2.3 Biochemical analyses

Blood was collected after 4 hr fasting by retro‐orbital puncture under isoflurane‐induced anesthesia and collected in BD microtainer (Cat. #365967, Becton, Dickinson and Company). Serum was separated by low‐speed centrifugation (3,000 rpm, 15 min, room temperature) and was stored in −80°C freezer.

### 2.4 Serum lipid measurements

TC, triglycerides (TGs), and HDL‐C levels were measured in duplicate in individual serum samples with commercially available kits (EKF Diagnostics‐Stanbio). Phospholipid (PL) concentrations were determined spectrophotometrically by commercially available kit (Wako Diagnostic).

### 2.5 HPLC separation of serum lipoprotein cholesterol and TGs

Fifty microliter of serum samples obtained on day 14 after OCA treatment or vehicle control from four mice of the same treatment group were pooled together and were analyzed for TG and cholesterol levels of each of the major lipoprotein classes including chylomicron (CM, >80 nm), very‐low density lipoprotein (VLDL; 30–80 nm), LDL (16–30 nm), and HDL (8–16 nm) by gel‐permeation high‐performance liquid chromatography (HPLC) system as described previously (Okazaki et al., 2005).

### 2.6 Hepatic and fecal lipids measurement

Frozen liver tissue (50 mg) and dried feces (30 mg) were homogenized in 1‐ml chloroform/methanol (2:1) mixture according to Folch method (Folch, Lees, & Sloane Stanley, 1957). Lipids were extracted by shaking samples for 16 hr at room temperature, followed by centrifugation at 5,000 rpm for 10 min. Supernatant was mixed with 0.2‐ml 0.9% saline and vortex for 20 s before centrifuged at 2000 rpm for 5 min. Lipids containing the lower phase was transferred into a siliconized microcentrifuge tube and air‐dried overnight under fume hood. Finally, dried lipids were dissolved in 0.25‐ml 10% Triton X‐100 containing isopropanol. TC, TG, and PL concentrations were estimated using commercial kits purchased from EKF Diagnostics‐Stanbio and Wako Diagnostic, respectively. Final lipid concentration was normalized per gram of liver weight or per gram of dried feces.

### 2.7 Measurement of fecal total BAs

BAs were measured enzymatically using the total BA kit (Cat. #DZ042A, Diazyme Laboratories Inc.). To determine the fecal BA excretion, the feces from individually housed mice were collected within 24‐hr period and dried. Then 0.5 g of dried feces was powdered using pestle and mortar and 30 mg of dried powder feces were used to extract BA in 75% ethanol at 50°C for 2 hr (Yu et al., 2000). BA concentration was measured enzymatically using the kit from Diazyme Laboratories.

### 2.8 Quantitative RT‐PCR

Total RNA was extracted from 15 to 20 mg of the liver and small intestine using RNeasy plus mini kit (Cat. #74136, Qiagen) according to the manufacturer's instructions. cDNA was synthesized from total RNA using the High Capacity cDNA Reverse Transcription Kits (Cat. #4368814, ThermoFisher Scientific). Quantitative real‐time PCR was conducted using PowerUp SYBR green master mix reagent (Cat. #A25742, ThermoFisher Scientific). QRT‐PCR amplification was performed in triplicates in a 384‐well plate for each cDNA sample on an ABI PRISM 7000 sequence detection system (Applied Biosystems). Target mRNA expression in each sample was normalized to the housekeeping gene GAPDH. The 2^‐ΔΔCt^ method was used to calculate relative mRNA expression levels. The primers used in this study are listed in Table 1.

**Table 1:** List of qRT‐PCR primer sequence

Gene names	Forward	Reverse
LDLR	ACCTGCCGACCTGATGAATTC	GCAGTCATGTTCACGGTCACA
SR‐B1	CTTGCTGCTGAGGGAGTCTCG	CTGAAGGAGACGGAGACAGAGG
SHP	CCGTGGAATGGAGTCTGG	CTTGCTGGACAGTTAGTAGTG
CYP7A1	GGGATTGCTGTGGTAGTGAGC	GGTATGGAATCAACCCGTTGTC
ABCA1	AACAGTTTGTGGCCCTTTTG	AGTTCCAGGCTGGCGTACTT
ABCG1	GGACTCGGTCCTGACACATC	CAGGTACAGCAGGCCAATGA
ABCG5	CCTGCAGAGCGACGTTTTTC	CCAATCATTTGGTCCGCCAC
ABCG8	CTGTGGAATGGGACTGTACTTC	GTTGGACTGACCACTGTAGGT
NPC1L1	TATACCCGTGGCCCCCATAA	CTGCTAGGCCCCCTTTAGGA
FGF‐15	GCCATCAAGGACGTCAGCA	CTTCCTCCGAGTAGCGAATCAG
GAPDH	ATGGTGAAGGTCGGTGTGAA	ACTGGAACATGTAGACCATGTAGT

### 2.9 Western blotting

Individual liver tissues (30–50 mg) were homogenized in a RIPA buffer supplemented with Protease Inhibitor Cocktail (Cat. #11697498001, Sigma) and Phosphatase Inhibitor Cocktail (Cat. #P2850, Sigma). Proteins were separated by SDS‐PAGE and immunoblotted with antibodies against SR‐B1 (Cat. #ab52629, Abcam), LDL receptor (LDLR; Cat. #3839‐100, Bio‐vision), CYP7A1 (Cat. # MABD42, Millipore‐Sigma), and β‐actin (Cat. #A1978, Sigma). Immunoblots were visualized using SuperSignal West Femto Chemiluminescent Substrate (Thermo Scientific) and a FluorChem E imaging system (ProteinSimple).

### 2.10 Statistical analysis

All data are reported as mean ± *SEM*. Data were analyzed by unpaired Student *t* test and one‐way ANOVA, followed by post hoc analysis. Tukey's multiple comparison posttest was performed to compare groups of four. Statistical significance was defined as *p < .05; **p < .01; ***p < .001.

## 3 RESULTS

### 3.1 Depletion of hepatic SR‐B1‐attenuated OCA‐mediated reduction of serum cholesterol in normolipidemic mice

**Figure 1:**
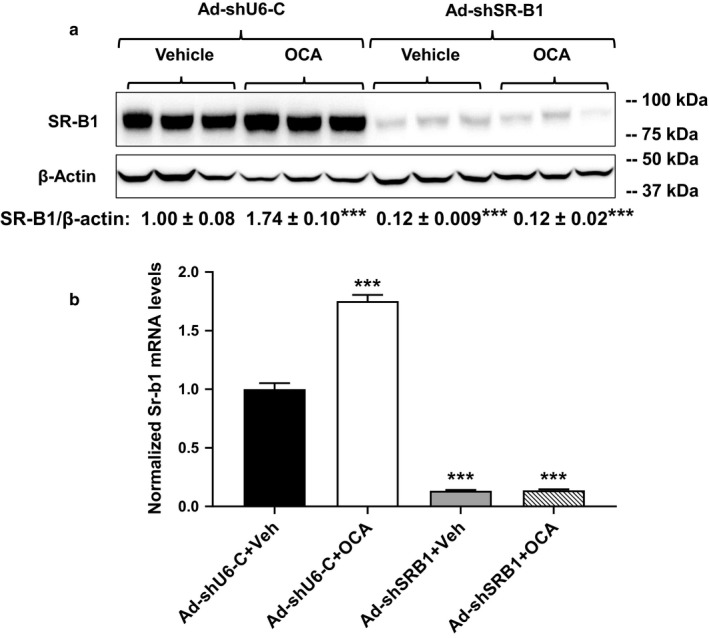
Depletion of hepatic SR‐B1 expression in adult mice fed a chow diet. Male C57BL/6J mice (*n* = 12 mice/group) were retro‐orbitally injected with 6 × 10^11^ IFU/mouse of Ad‐shU6 control or Ad‐shSR‐B1 adenovirus particles. Three days after injection, mice were orally treated with either OCA (40 mg/kg) or vehicle for 10 days. At termination of experiment, liver tissue was excised and protein and gene expression analyses performed. (a) Western blot analysis of SR‐B1 and β‐actin in liver tissue. The protein abundance of SR‐B1 was quantified with normalization by signals of ß‐actin using the Alpha View Software. Values are the mean ± *SEM* of six samples per group. (b) QRT‐PCR was used to determine the relative expression level of Sr‐b1 mRNA in liver tissue. Values are the mean ± *SEM* of six samples per group. Statistical significance was determined with one‐way ANOVA with Tukey's multiple comparisons test. ***p < .001 compared with the Ad‐shU6‐C treated with vehicle

To generate a liver‐specific SR‐B1‐deficient mouse model, we employed previously established shRNA technology (Singh, Kan, Kraemer, Sobel, & Liu, 2019) to deplete hepatic SR‐B1 using adenovirus expressing a mouse Sr‐b1 shRNA (Ad‐shSR‐B1). Mice fed a NCD were injected with Ad‐shSR‐B1 or Ad‐shU6‐C control virus. Three days after viral injection, mice were administered with either OCA (40 mg kg^−1^ day^−1^) or vehicle (0.5% CMC) for an additional 10 days. Measurement of hepatic protein and gene expression analysis by Western blot and qRT‐PCR showed an 88% (*p* < .001) reduction in hepatic SR‐B1 protein level as well as an 86.8% (*p* < .001) reduction in hepatic Sr‐b1 mRNA levels in Ad‐shSR‐B1‐injected mice compared to Ad‐shU6‐C‐injected control mice (Figure 1a,b). OCA treatment led to a 1.7‐fold (*p* < .001) increase in SR‐B1 protein and mRNA levels in liver tissue of control mice (Ad‐shU6‐C), consistent with previously published report (Papazyan et al., 2018). In contrast, OCA treatment did not change the hepatic SR‐B1 expression levels in mice injected with Ad‐shSR‐B1 (Figure 1a,b).

**Figure 2:**
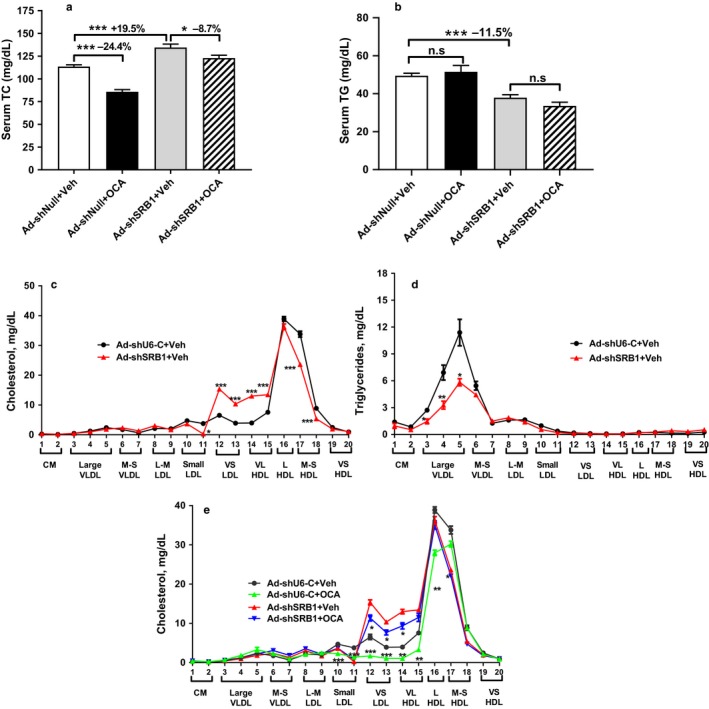
Activation of FXR by obeticholic acid (OCA) did not affect serum cholesterol levels in liver‐specific SR‐B1‐deficient mice fed an NCD. Three day after Ad‐shSR‐B1 or Ad‐shU6‐C adenovirus injection, mice were administered with either OCA or vehicle for additional 10 days. Four‐hours fasted serum samples were collected, and serum lipid levels were measured. (a) Serum TC, (b) serum TG. Values are the mean ± *SEM* of six samples per group. Statistical significance was determined with one‐way ANOVA with Tukey's multiple comparison posttest. ***p < .001 compared with the Ad‐shU6‐C mice treated with vehicle. Significance was determined by unpaired Student's *t* test; ^#^ *p* < .05 compared to Ad‐shSR‐B1 mice treated with vehicle. (c–e) Serum samples from two animals of the same treatment group were pooled together, and a total of three pooled serum samples from each group were analyzed for cholesterol (c, e) and triglyceride (d) distribution in HPLC‐separated lipoprotein factions. Data represent mean ± *SEM* of three samples per group. *p < .05; **p < .01; ***p < .001 compared with the Ad‐shU6‐C (c, d) without treatment or with OCA treatment (e)

Measurement of serum lipids showed that liver‐specific SR‐B1 depletion increased serum TC level by 19.5% (*p* < .001); OCA treatment reduced serum TC levels by 24.4% (*p* < .001) in mice injected with Ad‐shU6‐C, and this effect was attenuated in Ad‐shSR‐B1‐injected mice to only 8.7% reduction (Figure 2a). In addition, serum TG levels were slightly reduced in mice injected with Ad‐shSR‐B1 and OCA treatment did not affect TG levels in both groups (Figure 2b).

HPLC analysis further indicated that the increase in plasma cholesterol by SR‐B1 depletion was primarily due to increased quantities of very‐small low‐density lipoprotein (VSLDL), as well as in very‐large HDL particles (Figure 2c); this increase in HDL particle size by SR‐B1 deficiency was consistent with the previous published report (Huby et al., 2006). HPLC analysis of lipoprotein‐TG fractions revealed a reduction in VLDL‐TG by SR‐B1 depletion (Figure 2d). As shown in Figure 2e, OCA treatment in Ad‐shU6 control mice resulted in marked reductions in both HDL‐C as well as LDL‐C fractions; these reducing effects were less prominent in SR‐B1‐depleted mice.

### 3.2 FXR activation by OCA did not increase fecal cholesterol levels in liver‐specific SR‐B1‐deficient mice fed a chow diet

**Figure 3:**
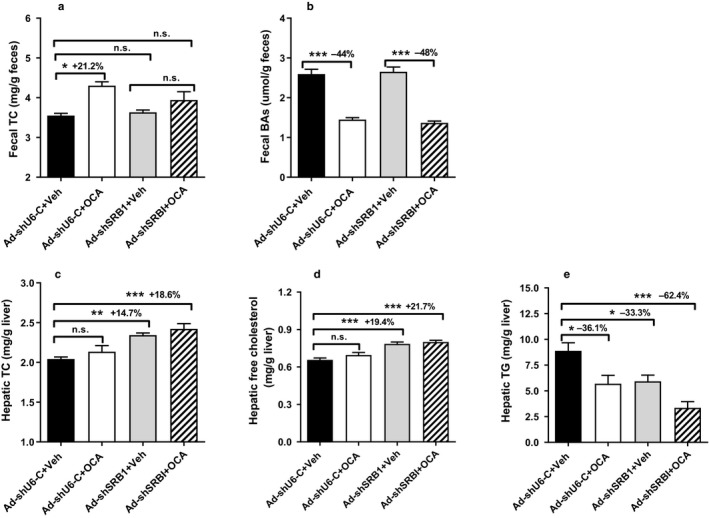
FXR activation by obeticholic acid (OCA) did not alter hepatic cholesterol content and fecal cholesterol levels in liver‐specific SR‐B1‐deficient mice fed a chow diet. Chow diet‐fed male black six mice were injected with Ad‐shU6 control or Ad‐shSR‐B1 adenovirus. Three days after adenovirus injection, mice were administered with OCA (40 mg/kg) or vehicle for 10 days. Feces were collected on day 0 and day 9 of treatment, dried, and weighed. Mice were euthanized, and liver and gallbladder were isolated at the termination of the experiment. Lipids were extracted and measured from individual liver or gallbladder samples. (a) Fecal TC, (b) fecal BA, (c‐e), hepatic TC, free cholesterol, and TG. Values are mean ± *SEM* of six samples per group. *p < .05 and ***p < .01 as compared with the Ad‐shU6 control or Ad‐shSR‐B1, OCA‐treated group

Next, we investigated the impact of hepatic SR‐B1 deficiency on fecal cholesterol and BA levels in untreated and OCA‐treated mice. Fecal cholesterol content was increased by OCA treatment in the control mice up to 21.2% of vehicle control, but this enhancing effect was not detected in mice injected with Ad‐shSR‐B1 (Figure 3a). As expected, reductions of fecal BA contents by OCA were detected in both the control and SR‐BI knockdown (KD) mice (Figure 3b). Hepatic lipid measurements showed that knocking down SR‐B1 in the liver increased hepatic TC by 14.7% of control (*p* < .01) and elevated free cholesterol (FC) abundance by 19.4% of control (*p* < .001) (Figure 3c,d). OCA treatment did not significantly change liver TC and FC contents in both the control and SR‐B1 KD mice fed a NCD. Hepatic TG contents were lower in Ad‐shSR‐B1‐injected mice and OCA treatment reduced liver TG contents in both groups (Figure 3e).

Collectively, these data together demonstrated that hepatic SR‐B1 deficiency abolished OCA‐induced transhepatic cholesterol excretion in mice fed a NCD, confirming the important role of hepatic SR‐B1 in transhepatic cholesterol movement under FXR activation.

### 3.3 Activation of FXR did not alter gene expression of cholesterol transporters in liver tissues but increased their expression in the ileum of hepatic SR‐B1‐deficient mice fed a chow diet

**Figure 4:**
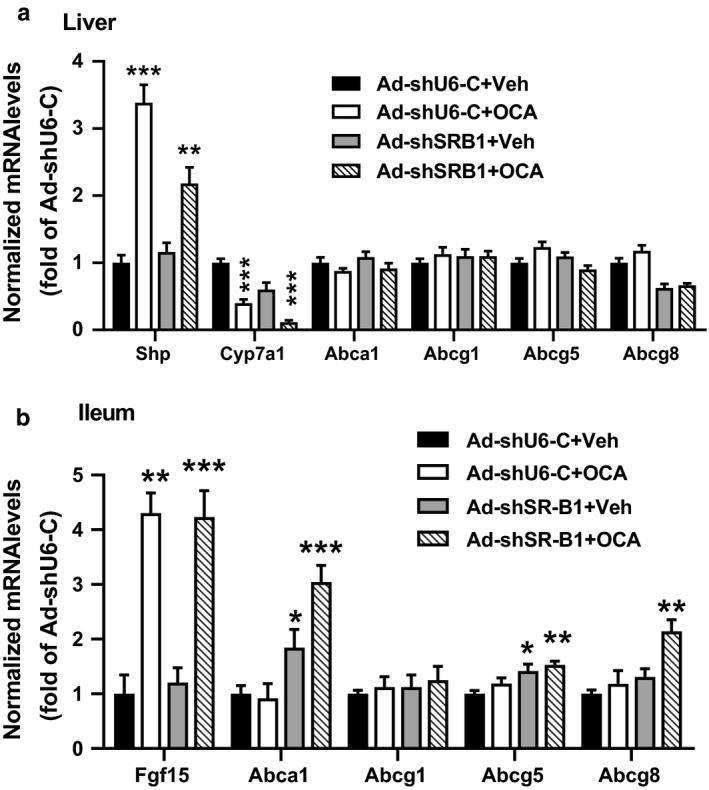
Activation of FXR did not alter the expression of transhepatic cholesterol transporter genes in liver‐specific SR‐B1‐deficient mice fed a chow diet. As described in Figures 1, 2, 3, the liver and ileum tissue were collected at the experimental termination, and qRT‐PCR was conducted to determine the relative expression levels of individual mRNAs after normalization with GAPDH mRNA levels. (a) Hepatic gene expression analysis, (b) ileum gene expression analysis. Statistical significance was determined with one‐way ANOVA with Tukey's multiple comparisons test. *p < .05; **p < .01; ***p < .001, compared with the Ad‐shU6‐C vehicle group, which was set at 1

Next, we investigated the influence of hepatic SR‐B1 deficiency on the expression of hepatic genes that are involved in BA synthesis and cholesterol efflux. Hepatic gene expression analysis by qRT‐PCR showed that mRNA levels of classical FXR target genes (Shp, Cyp7a1) were not affected by hepatic SR‐B1 depletion, and they were modulated by OCA treatment in both groups (Figure 4a). Hepatic Shp mRNA levels were induced 3.3‐fold in Ad‐shU6 control and 2.1‐fold in Ad‐shSR‐B1‐injected mice upon OCA treatment, and Cyp7a1 mRNA levels were substantially repressed by OCA in both groups, which was consistent with the significant reductions of BA contents in feces (Figure 3b). Hepatic mRNA levels of cholesterol transporters Abca1, Abcg1, Abcg5, and Abcg8 were not affected by either hepatic SR‐B1 depletion or OCA treatment.

Interestingly, gene expression analysis of ileum samples showed that hepatic SR‐B1 depletion significantly increased mRNA levels of Abca1 and Abcg5 in the ileum, and OCA treatment led to a further 1.5‐ to 2‐fold increase in mRNA level of Abca1 and Abcg8 in ileum samples of SR‐B1‐depleted mice as compared to the vehicle control. In addition, OCA treatment led to a 4‐fold (*p* < .001) increase in Fgf‐15 mRNA levels in the ileum of both groups (Figure 4b). Overall, these results indicate that activation of FXR by OCA in hepatic SR‐B1‐depleted mice under normolipidemic condition did not affect the gene expression of cholesterol transporters in the liver but modestly increased their expression levels in the intestine.

### 3.4 OCA treatment of NCD‐fed Sr‐b1 KO mice did not affect transhepatic cholesterol movement

**Figure 5:**
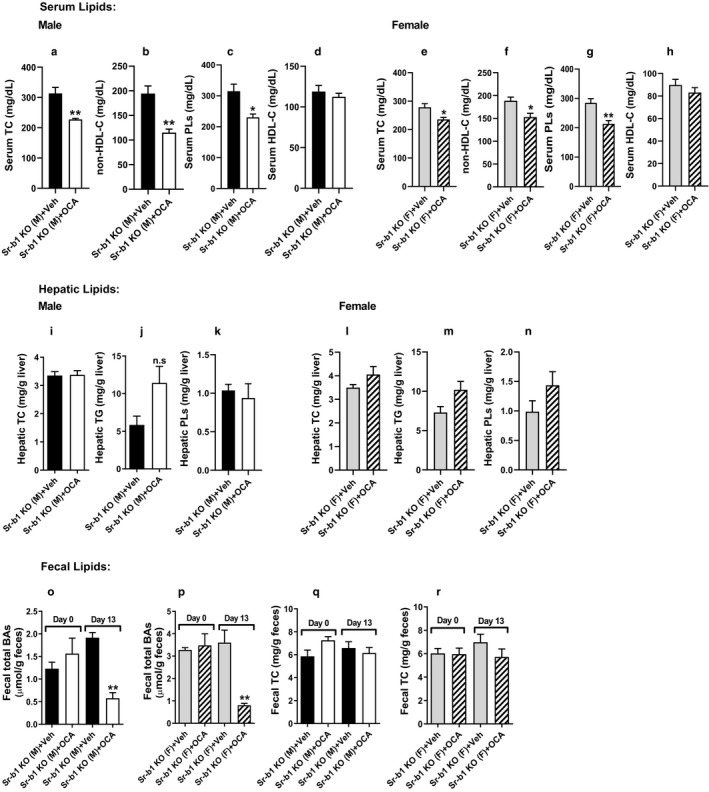
Obeticholic acid (OCA) reduced serum TC and non‐HDL‐C and did not affect hepatic and fecal cholesterol contents in normolipidemic Sr‐b1 KO mice. Chow diet‐fed male and female Sr‐b1 KO mice were administered with either OCA (40 mg/kg) or vehicle for 14 days. Serum lipid levels after treatment were measured (a‐h). Lipids and BA were also extracted and measured from liver (i‐n) and dried fecal samples (o‐r). Values are represented as mean ± *SEM*, *n* = 4/group. Statistical analysis was performed using Student's *t* test, n.s (nonsignificant), *p < .05 and **p < .01 as compared with vehicle group

To further investigate the impact of FXR activation on cholesterol metabolism under SR‐B1‐deficient condition, we utilized whole‐body Sr‐b1 KO mice fed a NCD. Male and female Sr‐b1 KO mice were treated with either OCA or vehicle for 14 days. OCA treatment did not change body weight and food intake (data not shown) but it modestly reduced serum TC levels and non‐HDL‐C levels in both male mice (Figure 5a,b) and female Sr‐b1 KO mice (Figure 5e,f). OCA treatment also lowered PL levels in all Sr‐b1 KO mice (Figure 5c,g). Importantly, serum HDL‐C levels were not affected by OCA treatments in these Sr‐b1 KO mice (Figure 5d,h). Moreover, hepatic TC, TG, and PL levels did not differ between control and OCA‐treated groups (Figure 5i–n). Analysis of fecal lipids showed that OCA treatment only reduced fecal BA contents (Figure 5o,p) in both male and female Sr‐b1 KO mice without any effects on fecal cholesterol amounts (Figure 5q,r). Collectively, these data demonstrated that the reduction of serum TC by OCA was not associated with transhepatic cholesterol excretion into feces.

**Figure 6:**
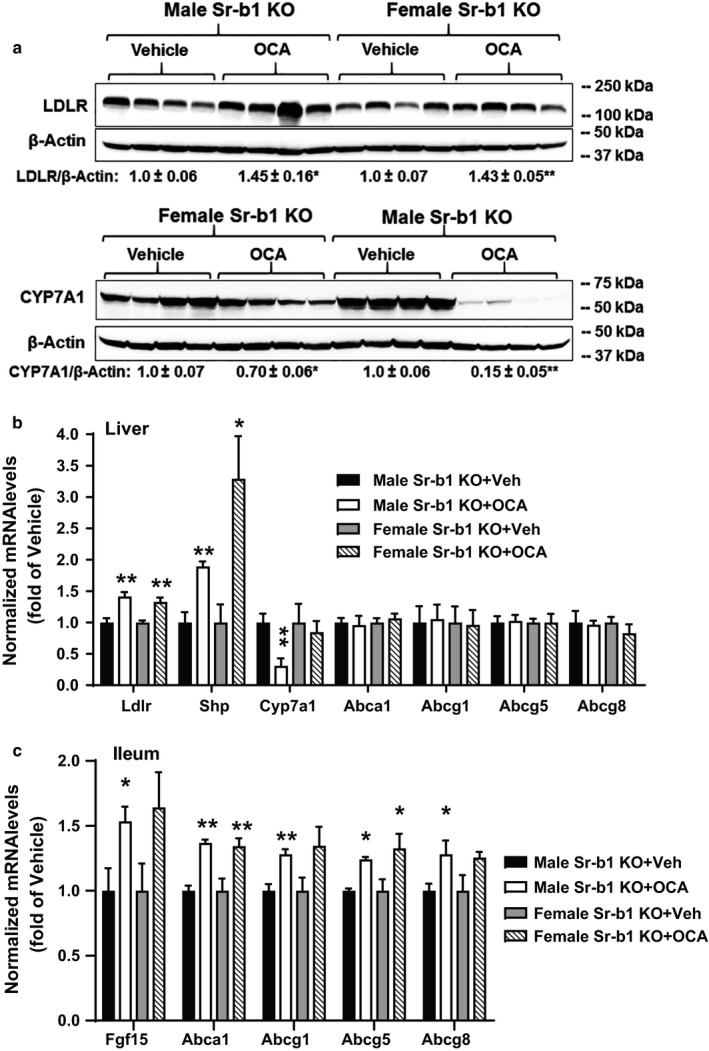
Obeticholic acid (OCA) does not alter transhepatic cholesterol efflux in Sr‐b1 KO mice fed a NCD. Seven to 8 weeks old male and female normolipidemic Sr‐b1 KO mice were administered with either OCA (40 mg/kg) or vehicle for 14 days. At the experiment termination, liver and ileum tissue were collected and protein and gene expression analyses performed. (a) Western blot analysis of LDLR, CYP7A1, and β‐Actin in liver tissue. Values are the mean ± *SEM* of four samples per group. *p < .05; ** *p* < .01 compared with the vehicle‐treated group. (b) Hepatic gene expression analysis by qRT‐PCR. (c) Ileum gene expression analysis. Statistical analysis was performed using Student's *t* test. *p < .05; **p < .01, compared with the vehicle group, which was set at 1

To gain a mechanistic insight into the OCA‐mediated reduction of serum TC and non‐HDL‐C levels in Sr‐b1 KO mice, we measured liver LDLR protein levels in all liver samples and the results showed that OCA treatment significantly increased hepatic LDLR protein level in both male (+45%, *p* < .05) and female (+43%, *p* < .01) Sr‐b1 KO mice fed a NCD (Figure 6a, upper panel). We also detected similar elevations of LDLR mRNA levels in male (+42%, *p* < .01) and female (+33%, *p* < .01) Sr‐b1 KO mice by OCA treatment (Figure 6b), which were consistent with our previous studies conducted in Sr‐b1 WT mice fed a NCD (Singh et al., 2018).

Analysis of hepatic gene expression of FXR target genes in BA synthesis and cholesterol transport pathways in the whole‐body Sr‐b1 KO mice showed the OCA‐induced elevation of Shp mRNA levels in both male and female mice and suppression of Cyp7a1 mRNA expression prominently in male Sr‐b1 KO mice. We further measured CYP7A1 protein levels in all liver samples (Figure 6a lower panel) that confirmed the different effects of OCA on Cyp7a1 expression between male and female mice. Like the hepatic depletion of SR‐B1, mRNA levels of cholesterol transporter genes in the liver did not differ between OCA‐ and vehicle‐treated Sr‐b1 KO mice. However, OCA treatment modestly elevated mRNA levels of Abca1, Abcg1, Abcg5, and Abcg8 in the ileum of these normolipidemic Sr‐b1 KO mice (Figure 6c), which is in line with the results of adenovirus‐mediated hepatic deletion of SR‐B1. Taken together, these results indicate that FXR activation by OCA in Sr‐b1 KO mice under a normolipidemic state does not affect transhepatic cholesterol efflux despite modest increases in cholesterol transporter genes in the ileum.

### 3.5 FXR activation substantially reduced serum cholesterol and triglycerides levels in Sr‐b1 KO mice fed a HFHCD

**Figure 7:**
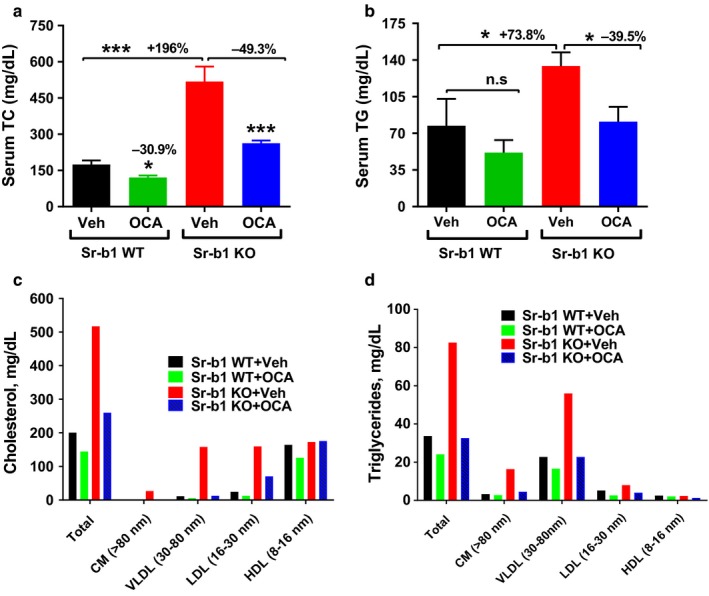
Obeticholic acid (OCA) treatment in hyperlipidemic Sr‐b1 KO mice diminishes serum cholesterol concentration. Male 8 weeks old Sr‐b1 KO and wild‐type littermates fed a HFHCD were administered with either OCA (40 mg/kg, *n* = 4) or vehicle (*n* = 4) for 14 days. At the experiment termination, 4‐h fasted serum samples were collected for the measurement of serum lipids. (a) Individual serum TC, (b) individual serum TG. Values are represented as mean ± *SEM*, *n* = 4 per group. Statistical significance was determined with one‐way ANOVA with Tukey's multiple comparison posttest. n.s (nonsignificant); *p < .05 and ***p < .001. Serum samples from four animals of the same treatment group were pooled together, and a total four pooled serum samples were analyzed for TC (c) and TG (d) distribution in HPLC‐separated lipoprotein factions

Next, we examined the impact of SR‐B1 deficiency on FXR‐regulated lipid metabolism under a hypercholesterolemic condition. Male Sr‐b1 KO mice and their WT littermates were fed a HFHCD for 2 weeks. While continuously on the HFHCD, mice were administered OCA or vehicle by oral gavage for 14 days. No obvious differences in food intake, body weight, or liver weight were observed between the OCA‐ and vehicle‐treated WT or Sr‐b1 KO mice (data not shown). Measurement of serum lipids showed that Sr‐b1 KO mice fed a HFHCD displayed higher serum TC (+196%, *p* < .001) and TG (+73.8%, *p* < .05) levels as compared to WT mice (Figure 7a,b). HPLC lipoprotein analysis of pooled serum showed that increased serum TC levels in Sr‐b1 KO mice were reflected by substantial elevations in the plasma concentration of VLDL‐C and LDL‐C. Surprisingly, OCA treatment led to a huge reduction in serum TC (−49.3%, *p* < .001) levels in HFHCD‐fed Sr‐b1 KO mice by significant reductions in VLDL and LDL‐C without any changes in HDL‐C (Figure 7c). In addition, OCA treatment reduced plasma levels of TG (−39.5, *p* < .05) mainly in VLDL‐TG fraction (Figure 7d). Compared to Sr‐b1 KO mice, OCA treatment in WT mice did not change serum TG levels and reduced serum TC levels by 30.9% (*p* < .05) as compared to vehicle control (Figure 7a). This reduction was reflected by a decrease in the HDL‐C fraction (Figure 7c) consistent with literature reports (Dong, Young, Liu, Singh, & Liu, 2017; Webb et al., 2002; Yancey et al., 2000).

### 3.6 OCA administration increased fecal cholesterol excretion in HFHCD‐fed Sr‐b1 KO mice

**Figure 8:**
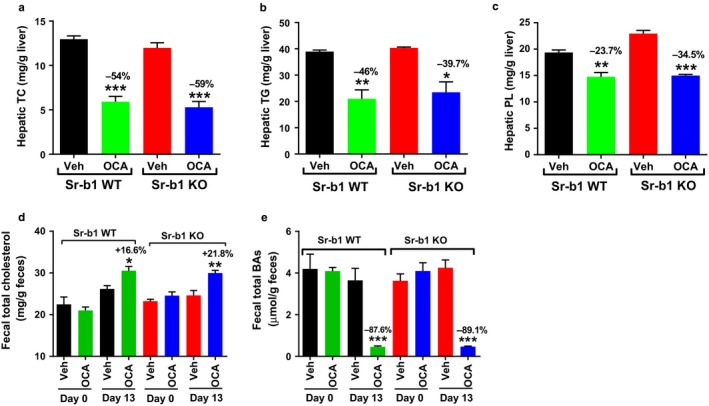
Obeticholic acid (OCA) administration in hyperlipidemic Sr‐b1 KO mice lowered hepatic cholesterol content and increases fecal cholesterol excretion. Male 8 weeks old Sr‐b1 KO and wild‐type littermates fed a HFHCD were administered with either OCA (40 mg/kg, *n* = 4) or vehicle (*n* = 4) for 14 days. Feces were collected on day 0 and day 13 of treatment, dried, and weighed. At the experiment termination, liver and gallbladder were collected from 4‐h fasted mice. Liver lipids were extracted and measured. (a) Hepatic TC, (b) hepatic TG, and (c) hepatic PL. Fecal BAs and cholesterol were extracted from dried feces. (d) Fecal total cholesterol, (e) fecal BAs. Values are represented as mean ± *SEM*, *n* = 4 mice/group, one‐way ANOVA with Tukey's multiple comparison posttest. *p < .05; **p < .01; ***p < .001

Hepatic cholesterol, TG, and PL contents did not differ between the WT and Sr‐b1 KO mice fed the HFHCD, and OCA treatment reduced their liver accumulations to similar extents (Figure 8a–c). Interestingly, fecal cholesterol contents in Sr‐b1 KO mice were increased 21.8% (*p* < .01) by OCA and that was even higher than its effect in WT mice (16.6%, *p* < .05) (Figure 8d). In addition, OCA treatment led to reductions of fecal BA amounts in both WT (−87.6%) and Sr‐b1 KO (−89.1%) mice (Figure 8e).

Taken together, these data demonstrate that under a hypercholesterolemic condition, FXR activation led to a substantial reduction of serum cholesterol and increased fecal cholesterol output in Sr‐b1 KO mice, suggesting that activated FXR might have utilized an alternative mechanism to eliminate cholesterol in the absence of SR‐B1, possibly through acceleration of biliary cholesterol secretion.

### 3.7 OCA treatment elevated the gene expression of intestinal cholesterol transporters in HFHCD‐fed Sr‐b1 KO mice

**Figure 9:**
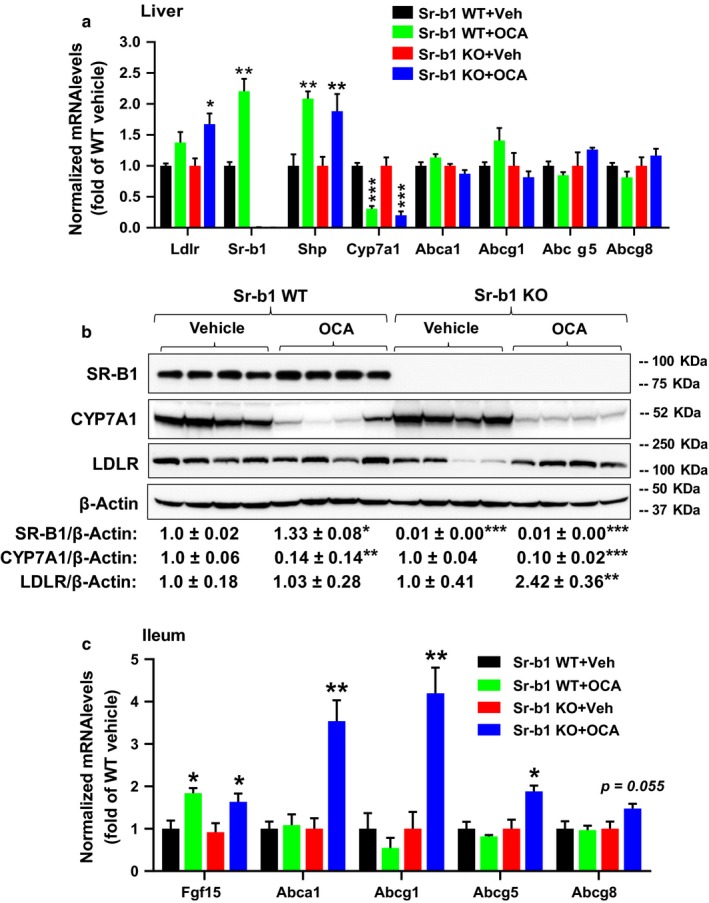
Obeticholic acid (OCA) treatment increased liver LDLR expression and stimulated the gene expression of cholesterol transporter in ileum. Male hyperlipidemic Sr‐b1 KO mice or wild‐type littermates were administered with either OCA (40 mg/kg) or vehicle for 14 days. At the experiment termination, liver and ileum tissue were excised and protein and gene expression analyses performed. (a) Hepatic gene expression analysis. (b) Western blot analysis of SR‐B1, LDLR, CYP7A1, and β‐Actin in liver tissue. (c) Ileum gene expression analysis. Values are the mean ± *SEM* of four samples per group. *p < .05; **p < .01; ***p < .001 compared with the vehicle‐ or OCA‐treated group. QRT‐PCR was conducted to determine the relative expression levels of individual mRNAs after normalization with GAPDH mRNA levels. (b) Statistical analysis was performed using Student's *t* test. *p < .05; **p < .0; ***p < .001, compared with the vehicle group, which was set at 1

Hepatic gene expression analysis showed that OCA treatment led to a 2‐fold increase (*p* < .01) in Sr‐b1 mRNA levels in the HFHCD‐fed WT mice (Figure 9a), which was further corroborated by the results of Western blotting of SR‐B1 protein of WT mice with a 33% increase over the control (Figure 9b). These results demonstrated that FXR activation by OCA upregulates hepatic SR‐B1 expression, which may account for the reduction of plasma HDL‐C and the increase in fecal cholesterol excretion in hyperlipidemic WT mice as previously reported (Trigatti, Rigotti, & Krieger, 2000). Hepatic gene expression analysis of BA metabolism revealed about 2‐fold increases (*p* < .01) in Shp mRNA levels and strong suppression of CYP7A1 protein and mRNA levels in OCA‐treated WT and Sr‐b1 KO mice (Figure 9a,b), which correlated with the reductions of BA concentration in feces.

Like the NCD‐fed mice, OCA did not affect the expression of cholesterol transporter genes Abca1, Abcg1, Abcg5, and Abcg8 in liver tissues of OCA‐treated WT and Sr‐b1 KO mice (Figure 9a). These results possibly exclude the involvement of hepatic cholesterol transporters in the elevated levels of biliary cholesterol secretion by OCA treatment.

Liver LDLR protein levels were increased 2.4‐fold (*p* < .01) (Figure 9b), and LDLR mRNA levels were increased about 50% by OCA treatment in Sr‐b1 KO mice (Figure 9a), but not in the liver of WT mice. These results indicated that the increase in liver LDLR amount in the hyperlipidemic Sr‐b1 KO mice contributed to the reduction in serum cholesterol by OCA.

Furthermore, gene expression analysis of ileum samples by qRT‐PCR detected 4‐fold increases in Abca1 and Abcg1 mRNA levels, 2‐fold increase in Abcg5 mRNA level, and a 50% increase in Abcg8 mRNA levels in OCA‐treated Sr‐b1 KO mice fed a HFHCD diet (Figure 9c), which was not observed in WT mice treated with OCA. These results suggest that in response to hyperlipidemia and in the absence of SR‐B1‐mediated transhepatic cholesterol movement, FXR activation could have elevated ileum cholesterol transporters (Abca1, Abcg1, Abcg5, and Abcg8) that led to enhanced cholesterol export from the enterocyte back into the lumen and excreted into feces.

## 4 DISCUSSION

SR‐B1 has been known not only to mediate HDL‐C uptake but also to play key roles in transhepatic cholesterol excretion. Several studies have demonstrated the link between FXR‐mediated plasma cholesterol reduction and the increase in hepatic SR‐B1 expression (Dong et al., 2017; Hambruch et al., 2012). However, the mechanism underlying the effects of FXR activation on transhepatic cholesterol excretion has not been investigated in SR‐B1‐deficient mice under a normolipidemic state or hyperlipidemic conditions.

To fill in this gap, we set out to investigate the importance of SR‐B1 in FXR‐regulated cholesterol and BA metabolism in mice fed a normal chow and a HFHCD. Our investigations lead to the following important new findings.

First, by utilizing adenovirus‐mediated gene KD, we showed that depletion of hepatic SR‐B1 in normolipidemic mice elevated serum TC levels and shifted HDL‐C particles to larger sizes. FXR activation by OCA effectively lowered serum TC in mice injected with the control virus (Ad‐sh‐U6C), but this effect was attenuated in Ad‐shSR‐B1‐transduced mice. Importantly, the OCA‐induced enhancement in fecal cholesterol excretion was completely abolished by Ad‐shSR‐B1 transduction. These results provided direct evidence for SR‐B1‐mediated and FXR‐induced enhancement in transhepatic cholesterol movement. Furthermore, we found that hepatic SR‐B1 deficiency and FXR activation in chow fed mice did not affect the expression of major genes involved in cholesterol flux (ABCA1, ABCG1, ABCG5, and ABCG8) in the liver (Figure 4a), but elevated the expression of Abca1, Abcg5, and Abcg8 to various extents in the ileum (Figure 4b).

Secondly, to follow‐up on these interesting observations, we utilized normolipidemic Sr‐b1 whole‐body KO mice and we examined the effects of OCA treatment on both male and female Sr‐b1 KO mice. In these animals, OCA treatment had no effects on HDL‐C levels, but reduced serum PL levels and modestly lowered serum TC and non‐HDL‐C levels (Figure 5a‐d). As we expected and consistent with the hepatic SR‐B1 KD, OCA did not increase fecal cholesterol amounts, but reduced fecal BA amounts in both genders. Our previous studies have demonstrated that OCA treatment reduces plasma TC in normolipidemic mice via a novel mechanism of upregulating hepatic LDLR by mRNA stabilization (Singh et al., 2018). In this study, we further detected increased LDLR protein and mRNA levels in both male and female Sr‐b1 KO mice fed a NCD (Figure 6a,b), which explained the modest reduction of serum TC and non‐HDL‐C in the absence of SR‐B1. Furthermore, we detected the specific induction of cholesterol transporter genes Abca1, Abcg1, Abcg5, and Abcg8 by OCA treatment in the ileum samples of both male and female Sr‐b1 KO mice without changing their expressions in liver tissues, which reinforced our findings made in Ad‐shSR‐B1‐transduced mice fed a NCD.

The selective inductions of cholesterol transporter genes in ileum but not in the liver by activated FXR in both hepatic SR‐B1‐depleted and whole‐body‐deficient mice were the most interesting new findings of this study up to this point. Since the expression levels of these cholesterol transporters have direct impacts on cholesterol absorption in intestine and on biliary cholesterol secretion, which ultimately affect cholesterol levels in the circulation, we conducted additional studies to examine the effects of OCA on cholesterol metabolism in Sr‐b1 KO mice and their WT littermates under a hyperlipidemic condition by feeding mice a HFHCD.

Unexpectedly, under hyperlipidemic conditions, we detected stronger reductions of serum TC and TG in Sr‐b1 KO mice than the WT mice after OCA treatment in the absence of HDL‐C changes (Figure 7). More impressively, OCA increased fecal cholesterol content by 21.8% of control (*p* < .01) in Sr‐b1 KO mice higher than its effect in WT mice (16.6%, *p* < .05), which is in contrast to the lack of changes in fecal cholesterol in OCA‐treated Sr‐b1 KO mice under a normolipidemic condition (Figure 5q,r).

Hepatic gene expression analysis revealed that LDLR mRNA level was increased by OCA in Sr‐b1 KO mice and not in the WT mice, whereas Sr‐b1 mRNA levels were increased 2.2‐fold by OCA in the WT mice (Figure 9a). Western blot analysis of LDLR and SR‐B1 corroborated the qRT‐PCR results (Figure 9b). Thus, the increased LDLR expression likely contributed to the reduction of serum TC and non‐HDL cholesterol lipoprotein fractions in Sr‐b1 KO mice to some degree. Previous studies have shown that hepatic SR‐B1 expression increases RCT (Zhang et al., 2005) and is associated with biliary cholesterol secretion (Wiersma, Gatti, Nijstad, Kuipers, & Tietge, 2009). In agreement with this, our results indicate that increased hepatic SR‐B1 expression in WT mice by OCA directly correlated with reduction in circulating plasma cholesterol concentration as well as an increase in fecal cholesterol excretion.

It has been previously proposed that increased expression of Abca1 in the intestine reduces the efficacy of intestinal cholesterol absorption and enhances fecal cholesterol disposal (Repa et al., 2000). In addition, expression levels of Abcg5 and Abcg8 are implicated in control of cholesterol absorption (Berge et al., 2000), and it was shown that overexpression of human ABCG5/ABCG8 in mice increased gallbladder cholesterol concentration and fecal neutral sterol excretion (Yu et al., 2002). Interestingly, we detected an approximately 4‐fold increase in Abca1 and Abcg1 mRNA levels, a 2‐fold increase in Abcg5 mRNA, and 50% increase in Abcg8 mRNA levels only in Sr‐b1 KO mice, but not in the WT mice under the HFHCD‐fed condition. Thus, we speculate that the increased expression of these intestinal cholesterol transporters in Sr‐b1 KO mice by OCA treatment may contribute to the reduced plasma cholesterol and increased fecal cholesterol in addition to the improved removal of LDL‐C via OCA‐induced upregulation of LDLR.

In summary, we have demonstrated that under a hyperlipidemic state, in the absence of SR‐B1, activation of FXR promoted intestinal cholesterol excretion and diminished hyperlipidemia possibly by increasing the expression of cholesterol transporters in ileum to compensate for the loss of SR‐B1‐mediated transhepatic cholesterol movement in mice. It is important to note that regulations of plasma cholesterol metabolism by FXR agonists in humans are different than that in rodents. In nonalcoholic steatohepatitis patients, OCA treatment increases serum LDL‐C and TC levels. Future studies are needed to validate the findings demonstrated in this study to further understand the impact of FXR activation in intestine on plasma cholesterol metabolism and on the risk of coronary heart disease.

## CONFLICT OF INTEREST

None of authors have a conflict of interest regarding this study.
